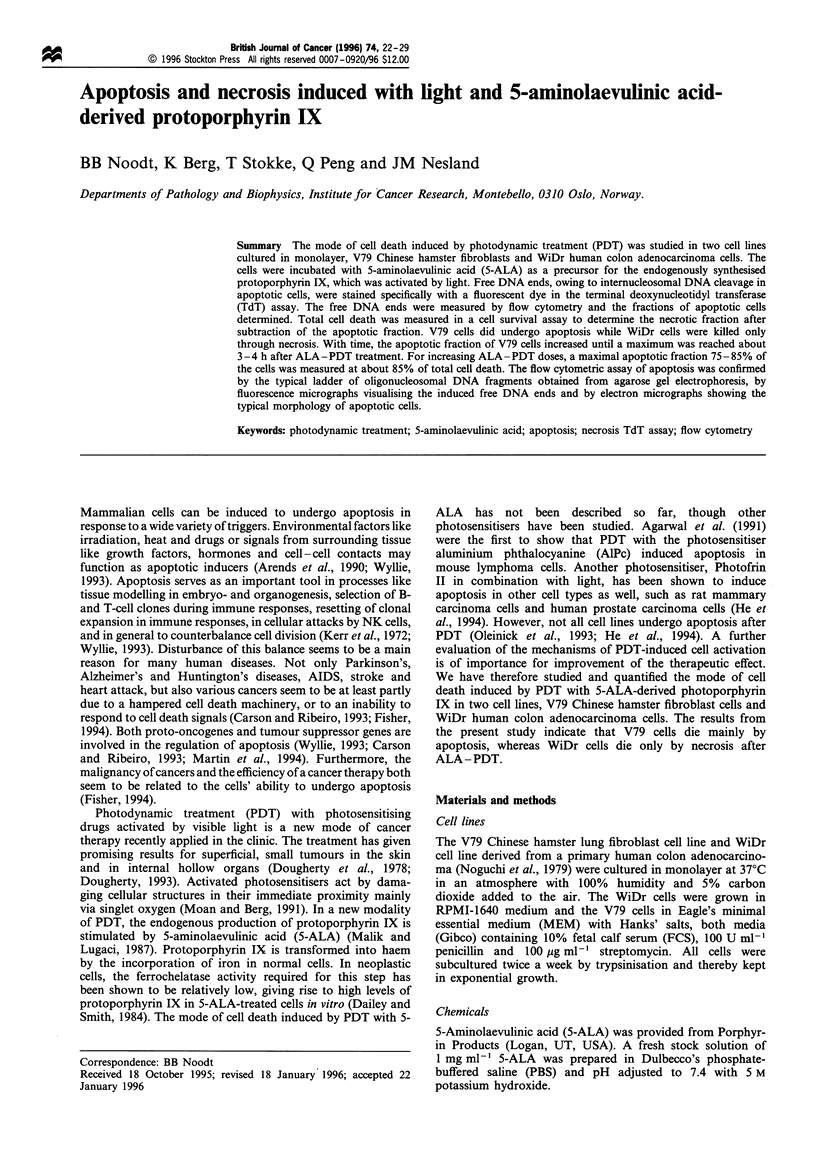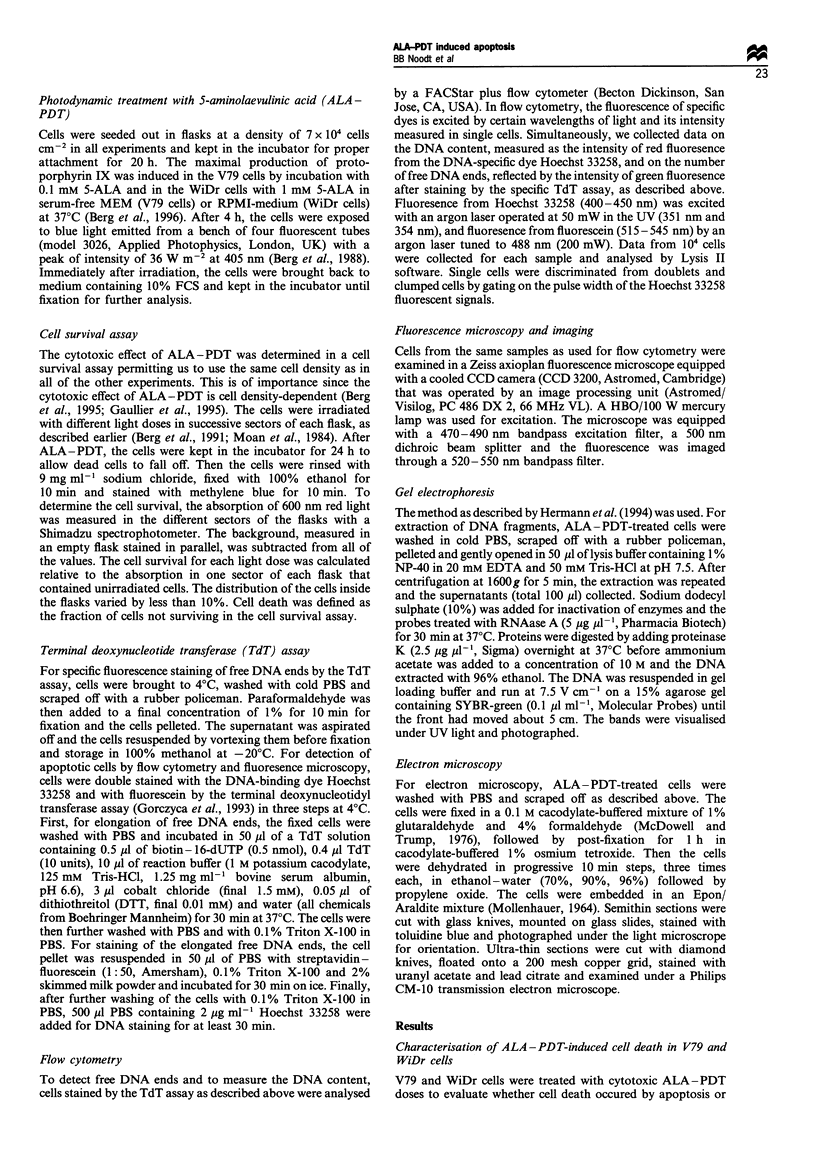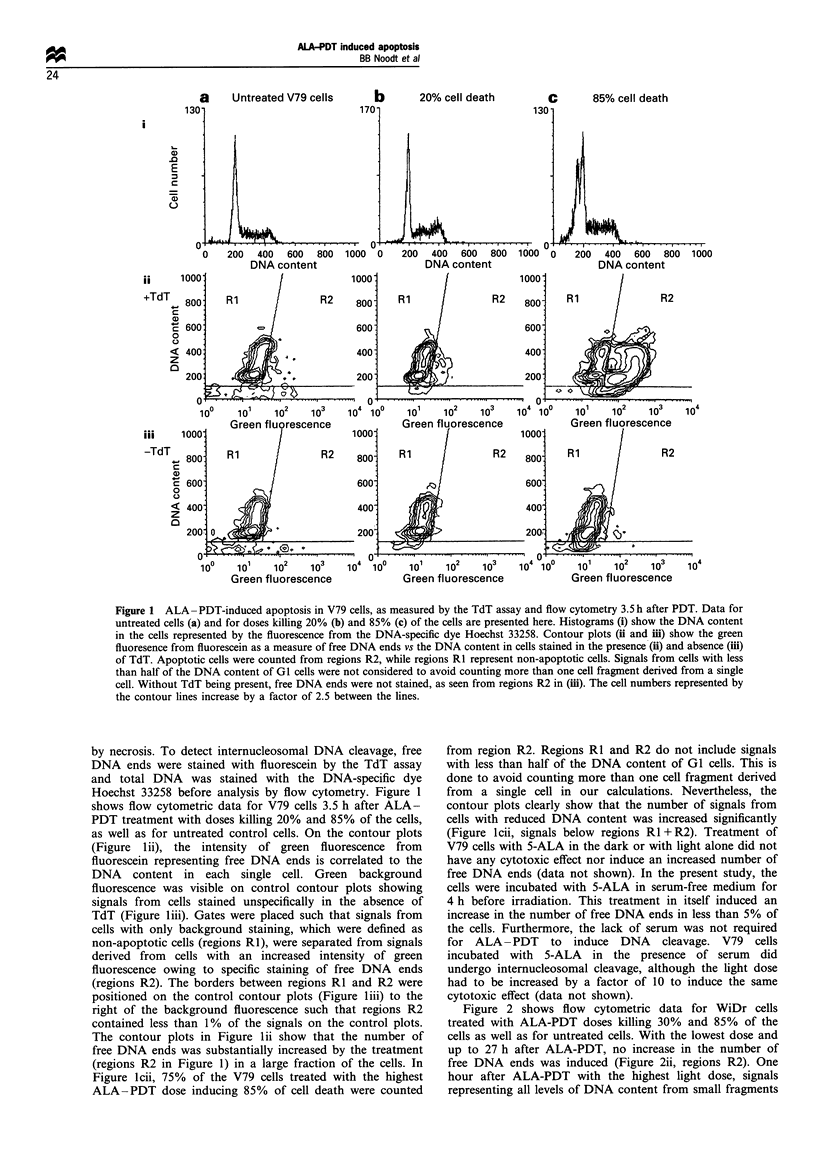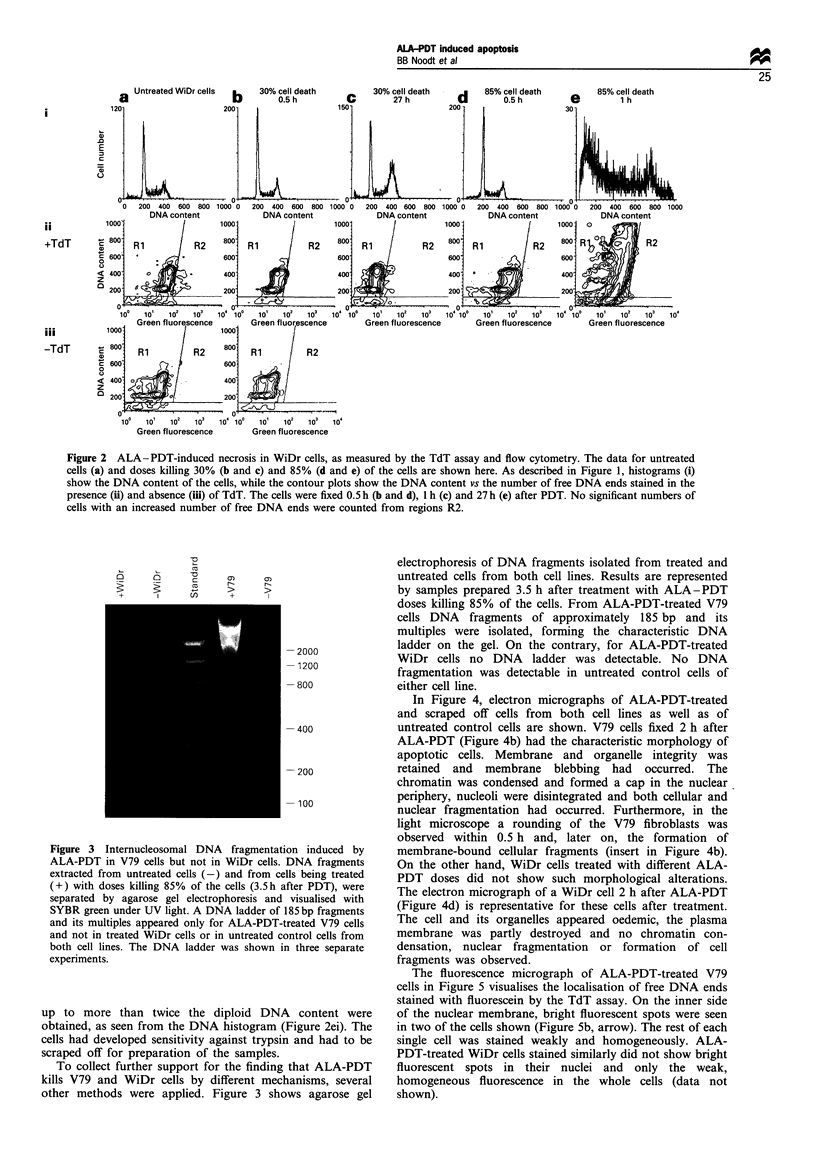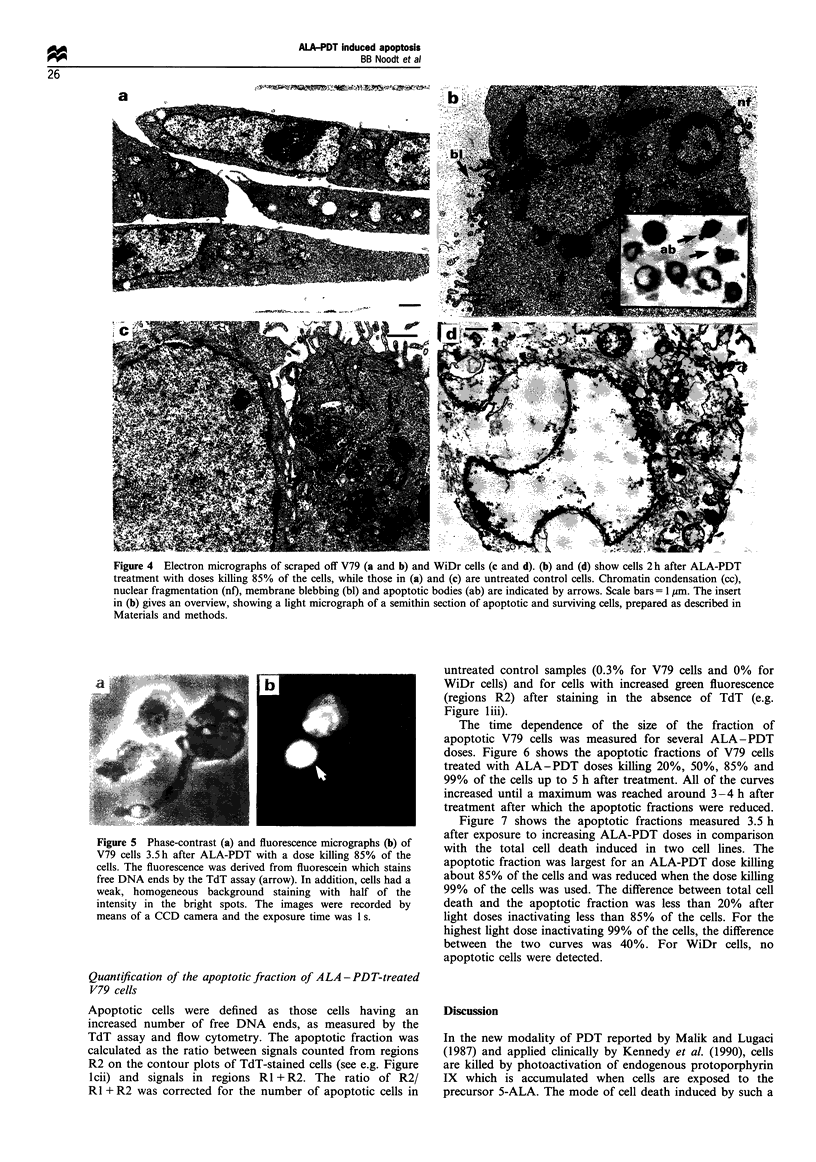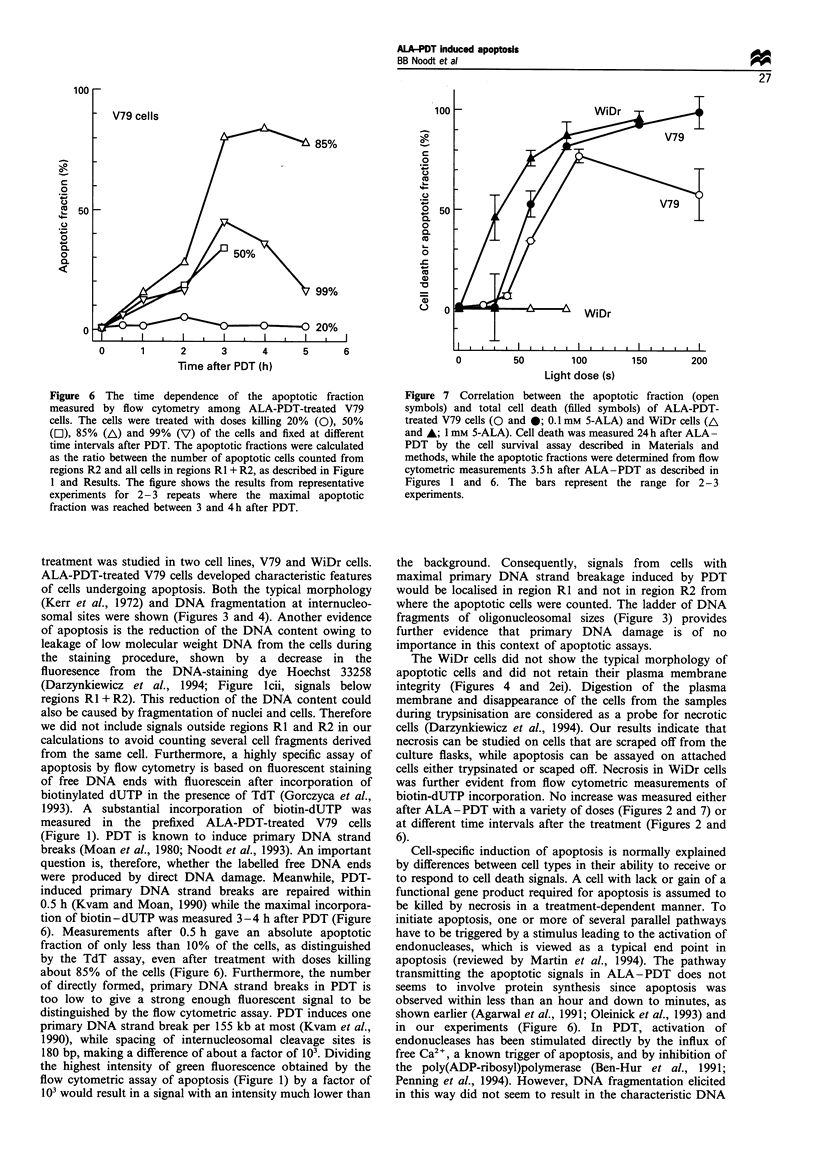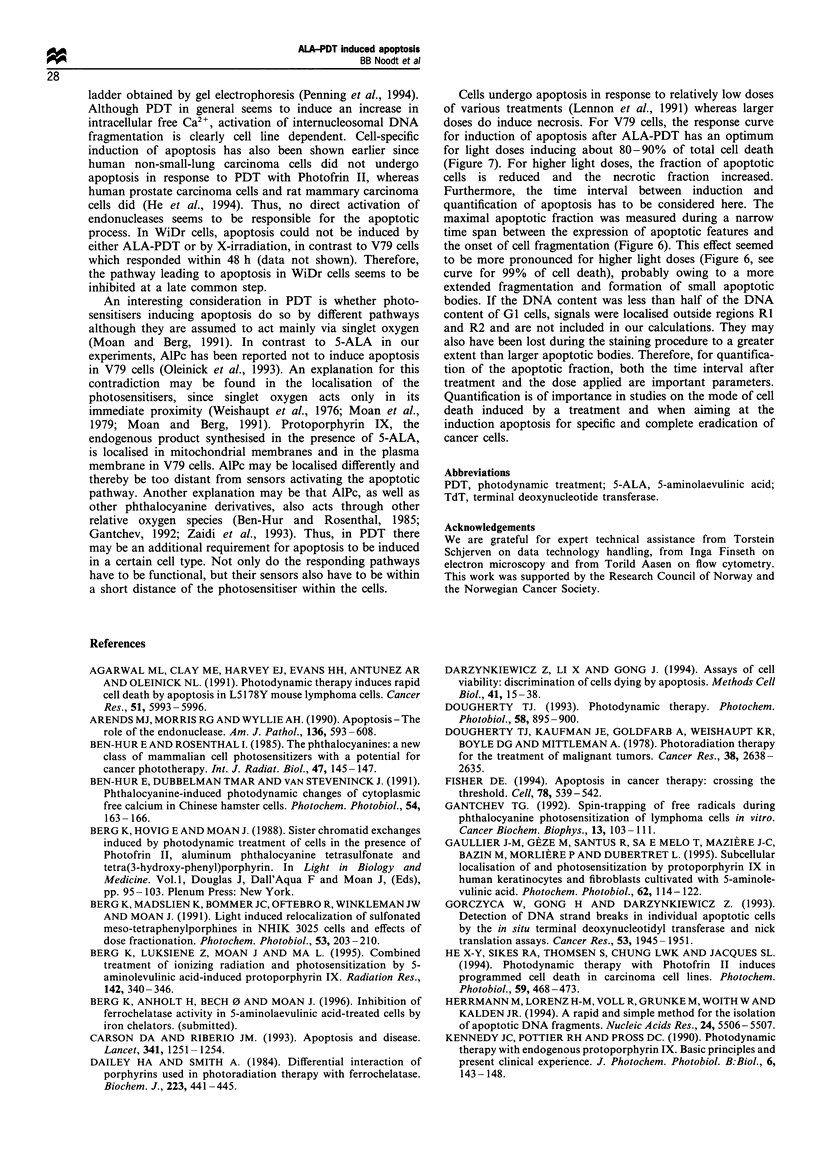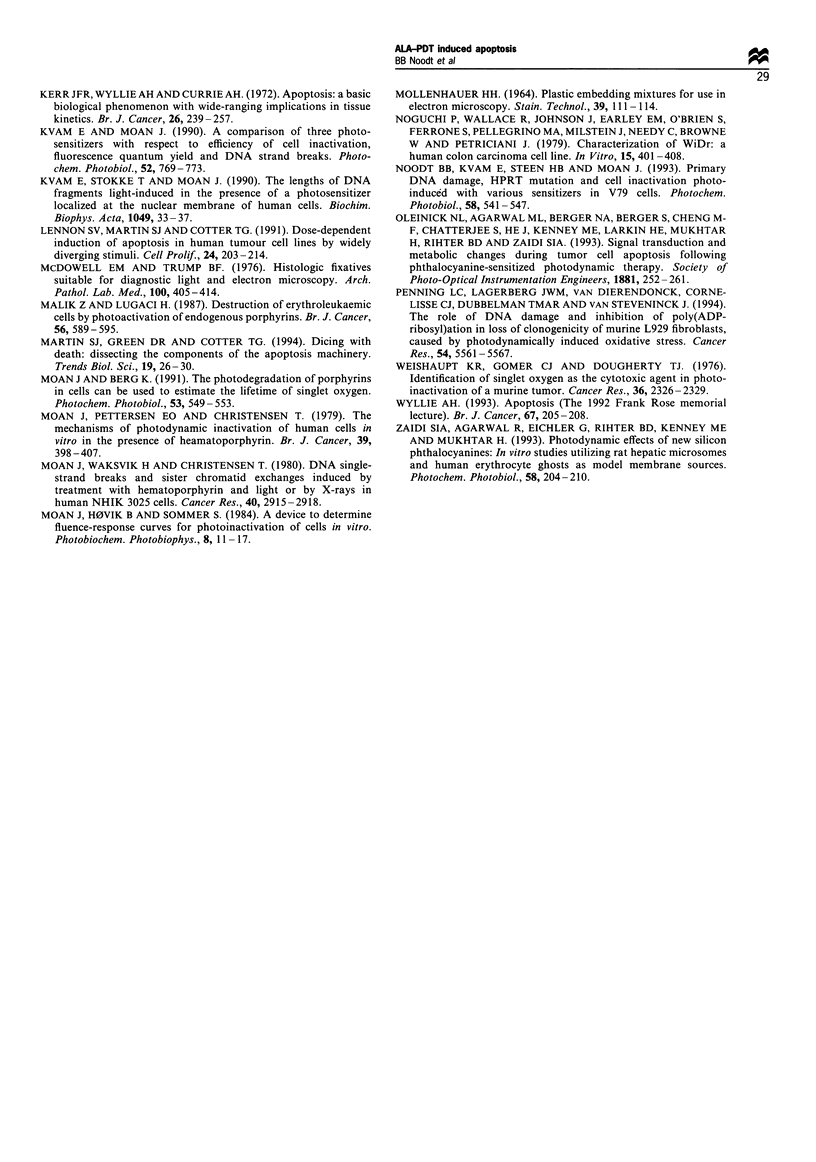# Apoptosis and necrosis induced with light and 5-aminolaevulinic acid-derived protoporphyrin IX.

**DOI:** 10.1038/bjc.1996.310

**Published:** 1996-07

**Authors:** B. B. Noodt, K. Berg, T. Stokke, Q. Peng, J. M. Nesland

**Affiliations:** Department of Pathology, Institute for Cancer Research, Montebello, Oslo, Norway.

## Abstract

**Images:**


British Journal of Cancer (1996) 74, 22-29
© 1996 Stockton Press All rights reserved 0007-0920/96 $12.00

### Apoptosis and necrosis induced with light and 5-aminolaevulinic acid-derived protoporphyrin IX

BB Noodt, K Berg, T Stokke, Q Peng and JM Nesland

*Departments of Pathology and Biophysics, Institute for Cancer Research, Montebello, 0310 Oslo, Norway.*

**Summary** The mode of cell death induced by photodynamic treatment (PDT) was studied in two cell lines cultured in monolayer, V79 Chinese hamster fibroblasts and WiDr human colon adenocarcinoma cells. The cells were incubated with 5-aminolaevulinic acid (5-ALA) as a precursor for the endogenously synthesised protoporphyrin IX, which was activated by light. Free DNA ends, owing to internucleosomal DNA cleavage in apoptotic cells, were stained specifically with a fluorescent dye in the terminal deoxynucleotidyl transferase (TdT) assay. The free DNA ends were measured by flow cytometry and the fractions of apoptotic cells determined. Total cell death was measured in a cell survival assay to determine the necrotic fraction after subtraction of the apoptotic fraction. V79 cells did undergo apoptosis while WiDr cells were killed only through necrosis. With time, the apoptotic fraction of V79 cells increased until a maximum was reached about 3-4 h after ALA-PDT treatment. For increasing ALA-PDT doses, a maximal apoptotic fraction 75-85% of the cells was measured at about 85% of total cell death. The flow cytometric assay of apoptosis was confirmed by the typical ladder of oligonucleosomal DNA fragments obtained from agarose gel electrophoresis, by fluorescence micrographs visualising the induced free DNA ends and by electron micrographs showing the typical morphology of apoptotic cells.

**Keywords:** photodynamic treatment; 5-aminolaevulinic acid; apoptosis; necrosis TdT assay; flow cytometry

Mammalian cells can be induced to undergo apoptosis in response to a wide variety of triggers. Environmental factors like irradiation, heat and drugs or signals from surrounding tissue like growth factors, hormones and cell-cell contacts may function as apoptotic inducers (Arends *et al.*, 1990; Wyllie, 1993). Apoptosis serves as an important tool in processes like tissue modelling in embryo- and organogenesis, selection of B- and T-cell clones during immune responses, resetting of clonal expansion in immune responses, in cellular attacks by NK cells, and in general to counterbalance cell division (Kerr *et al.*, 1972; Wyllie, 1993). Disturbance of this balance seems to be a main reason for many human diseases. Not only Parkinson's, Alzheimer's and Huntington's diseases, AIDS, stroke and heart attack, but also various cancers seem to be at least partly due to a hampered cell death machinery, or to an inability to respond to cell death signals (Carson and Ribeiro, 1993; Fisher, 1994). Both proto-oncogenes and tumour suppressor genes are involved in the regulation of apoptosis (Wyllie, 1993; Carson and Ribeiro, 1993; Martin *et al.*, 1994). Furthermore, the malignancy of cancers and the efficiency of a cancer therapy both seem to be related to the cells' ability to undergo apoptosis (Fisher, 1994).

Photodynamic treatment (PDT) with photosensitising drugs activated by visible light is a new mode of cancer therapy recently applied in the clinic. The treatment has given promising results for superficial, small tumours in the skin and in internal hollow organs (Dougherty *et al.*, 1978; Dougherty, 1993). Activated photosensitisers act by damaging cellular structures in their immediate proximity mainly via singlet oxygen (Moan and Berg, 1991). In a new modality of PDT, the endogenous production of protoporphyrin IX is stimulated by 5-aminolaevulinic acid (5-ALA) (Malik and Lugaci, 1987). Protoporphyrin IX is transformed into haem by the incorporation of iron in normal cells. In neoplastic cells, the ferrochelatase activity required for this step has been shown to be relatively low, giving rise to high levels of protoporphyrin IX in 5-ALA-treated cells *in vitro* (Dailey and Smith, 1984). The mode of cell death induced by PDT with 5-ALA has not been described so far, though other photosensitisers have been studied. Agarwal *et al.* (1991) were the first to show that PDT with the photosensitiser aluminium phthalocyanine (AlPc) induced apoptosis in mouse lymphoma cells. Another photosensitiser, Photofrin II in combination with light, has been shown to induce apoptosis in other cell types as well, such as rat mammary carcinoma cells and human prostate carcinoma cells (He *et al.*, 1994). However, not all cell lines undergo apoptosis after PDT (Oleinick *et al.*, 1993; He *et al.*, 1994). A further evaluation of the mechanisms of PDT-induced cell activation is of importance for improvement of the therapeutic effect. We have therefore studied and quantified the mode of cell death induced by PDT with 5-ALA-derived photoporphyrin IX in two cell lines, V79 Chinese hamster fibroblast cells and WiDr human colon adenocarcinoma cells. The results from the present study indicate that V79 cells die mainly by apoptosis, whereas WiDr cells die only by necrosis after ALA-PDT.

### Materials and methods

### *Cell lines*

The V79 Chinese hamster lung fibroblast cell line and WiDr cell line derived from a primary human colon adenocarcinoma (Noguchi *et al.*, 1979) were cultured in monolayer at 37°C in an atmosphere with 100% humidity and 5% carbon dioxide added to the air. The WiDr cells were grown in RPMI-1640 medium and the V79 cells in Eagle's minimal essential medium (MEM) with Hanks' salts, both media (Gibco) containing 10% fetal calf serum (FCS), 100 U $ml^{-1}$ penicillin and 100 $\mu g\ ml^{-1}$ streptomycin. All cells were subcultured twice a week by trypsinisation and thereby kept in exponential growth.

### *Chemicals*

5-Aminolaevulinic acid (5-ALA) was provided from Porphyrin Products (Logan, UT, USA). A fresh stock solution of 1 mg $ml^{-1}$ 5-ALA was prepared in Dulbecco's phosphate-buffered saline (PBS) and pH adjusted to 7.4 with 5 M potassium hydroxide.

Correspondence: BB Noodt
Received 18 October 1995; revised 18 January 1996; accepted 22 January 1996

### *Photodynamic treatment with 5-aminolaevulinic acid (ALA–PDT)*

Cells were seeded out in flasks at a density of $7 \times 10^4$ cells $cm^{-2}$ in all experiments and kept in the incubator for proper attachment for 20 h. The maximal production of protoporphyrin IX was induced in the V79 cells by incubation with 0.1 mM 5-ALA and in the WiDr cells with 1 mM 5-ALA in serum-free MEM (V79 cells) or RPMI-medium (WiDr cells) at 37°C (Berg *et al.*, 1996). After 4 h, the cells were exposed to blue light emitted from a bench of four fluorescent tubes (model 3026, Applied Photophysics, London, UK) with a peak of intensity of 36 W $m^{-2}$ at 405 nm (Berg *et al.*, 1988). Immediately after irradiation, the cells were brought back to medium containing 10% FCS and kept in the incubator until fixation for further analysis.

### *Cell survival assay*

The cytotoxic effect of ALA–PDT was determined in a cell survival assay permitting us to use the same cell density as in all of the other experiments. This is of importance since the cytotoxic effect of ALA–PDT is cell density-dependent (Berg *et al.*, 1995; Gaullier *et al.*, 1995). The cells were irradiated with different light doses in successive sectors of each flask, as described earlier (Berg *et al.*, 1991; Moan *et al.*, 1984). After ALA–PDT, the cells were kept in the incubator for 24 h to allow dead cells to fall off. Then the cells were rinsed with 9 mg $ml^{-1}$ sodium chloride, fixed with 100% ethanol for 10 min and stained with methylene blue for 10 min. To determine the cell survival, the absorption of 600 nm red light was measured in the different sectors of the flasks with a Shimadzu spectrophotometer. The background, measured in an empty flask stained in parallel, was subtracted from all of the values. The cell survival for each light dose was calculated relative to the absorption in one sector of each flask that contained unirradiated cells. The distribution of the cells inside the flasks varied by less than 10%. Cell death was defined as the fraction of cells not surviving in the cell survival assay.

### *Terminal deoxynucleotide transferase (TdT) assay*

For specific fluorescence staining of free DNA ends by the TdT assay, cells were brought to 4°C, washed with cold PBS and scraped off with a rubber policeman. Paraformaldehyde was then added to a final concentration of 1% for 10 min for fixation and the cells pelleted. The supernatant was aspirated off and the cells resuspended by vortexing them before fixation and storage in 100% methanol at −20°C. For detection of apoptotic cells by flow cytometry and fluoresence microscopy, cells were double stained with the DNA-binding dye Hoechst 33258 and with fluorescein by the terminal deoxynucleotidyl transferase assay (Gorczyca *et al.*, 1993) in three steps at 4°C. First, for elongation of free DNA ends, the fixed cells were washed with PBS and incubated in 50 $\mu l$ of a TdT solution containing 0.5 $\mu l$ of biotin–16-dUTP (0.5 nmol), 0.4 $\mu l$ TdT (10 units), 10 $\mu l$ of reaction buffer (1 M potassium cacodylate, 125 mM Tris-HCl, 1.25 mg $ml^{-1}$ bovine serum albumin, pH 6.6), 3 $\mu l$ cobalt chloride (final 1.5 mM), 0.05 $\mu l$ of dithiothreitol (DTT, final 0.01 mM) and water (all chemicals from Boehringer Mannheim) for 30 min at 37°C. The cells were then further washed with PBS and with 0.1% Triton X-100 in PBS. For staining of the elongated free DNA ends, the cell pellet was resuspended in 50 $\mu l$ of PBS with streptavidin–fluorescein (1:50, Amersham), 0.1% Triton X-100 and 2% skimmed milk powder and incubated for 30 min on ice. Finally, after further washing of the cells with 0.1% Triton X-100 in PBS, 500 $\mu l$ PBS containing 2 $\mu g$ $ml^{-1}$ Hoechst 33258 were added for DNA staining for at least 30 min.

### *Flow cytometry*

To detect free DNA ends and to measure the DNA content, cells stained by the TdT assay as described above were analysed by a FACStar plus flow cytometer (Becton Dickinson, San Jose, CA, USA). In flow cytometry, the fluorescence of specific dyes is excited by certain wavelengths of light and its intensity measured in single cells. Simultaneously, we collected data on the DNA content, measured as the intensity of red fluoresence from the DNA-specific dye Hoechst 33258, and on the number of free DNA ends, reflected by the intensity of green fluoresence after staining by the specific TdT assay, as described above. Fluoresence from Hoechst 33258 (400–450 nm) was excited with an argon laser operated at 50 mW in the UV (351 nm and 354 nm), and fluoresence from fluorescein (515–545 nm) by an argon laser tuned to 488 nm (200 mW). Data from $10^4$ cells were collected for each sample and analysed by Lysis II software. Single cells were discriminated from doublets and clumped cells by gating on the pulse width of the Hoechst 33258 fluorescent signals.

### *Fluorescence microscopy and imaging*

Cells from the same samples as used for flow cytometry were examined in a Zeiss axioplan fluorescence microscope equipped with a cooled CCD camera (CCD 3200, Astromed, Cambridge) that was operated by an image processing unit (Astromed/Visilog, PC 486 DX 2, 66 MHz VL). A HBO/100 W mercury lamp was used for excitation. The microscope was equipped with a 470–490 nm bandpass excitation filter, a 500 nm dichroic beam splitter and the fluorescence was imaged through a 520–550 nm bandpass filter.

### *Gel electrophoresis*

The method as described by Hermann *et al.* (1994) was used. For extraction of DNA fragments, ALA–PDT-treated cells were washed in cold PBS, scraped off with a rubber policeman, pelleted and gently opened in 50 $\mu l$ of lysis buffer containing 1% NP-40 in 20 mM EDTA and 50 mM Tris-HCl at pH 7.5. After centrifugation at 1600$g$ for 5 min, the extraction was repeated and the supernatants (total 100 $\mu l$) collected. Sodium dodecyl sulphate (10%) was added for inactivation of enzymes and the probes treated with RNAase A (5 $\mu g$ $\mu l^{-1}$, Pharmacia Biotech) for 30 min at 37°C. Proteins were digested by adding proteinase K (2.5 $\mu g$ $\mu l^{-1}$, Sigma) overnight at 37°C before ammonium acetate was added to a concentration of 10 M and the DNA extracted with 96% ethanol. The DNA was resuspended in gel loading buffer and run at 7.5 V $cm^{-1}$ on a 15% agarose gel containing SYBR-green (0.1 $\mu l$ $ml^{-1}$, Molecular Probes) until the front had moved about 5 cm. The bands were visualised under UV light and photographed.

### *Electron microscopy*

For electron microscopy, ALA–PDT-treated cells were washed with PBS and scraped off as described above. The cells were fixed in a 0.1 M cacodylate-buffered mixture of 1% glutaraldehyde and 4% formaldehyde (McDowell and Trump, 1976), followed by post-fixation for 1 h in cacodylate-buffered 1% osmium tetroxide. Then the cells were dehydrated in progressive 10 min steps, three times each, in ethanol–water (70%, 90%, 96%) followed by propylene oxide. The cells were embedded in an Epon/Araldite mixture (Mollenhauer, 1964). Semithin sections were cut with glass knives, mounted on glass slides, stained with toluidine blue and photographed under the light microscrope for orientation. Ultra-thin sections were cut with diamond knives, floated onto a 200 mesh copper grid, stained with uranyl acetate and lead citrate and examined under a Philips CM-10 transmission electron microscope.

### Results

### *Characterisation of ALA–PDT-induced cell death in V79 and WiDr cells*

V79 and WiDr cells were treated with cytotoxic ALA–PDT doses to evaluate whether cell death occured by apoptosis or

**Figure 1** ALA-PDT-induced apoptosis in V79 cells, as measured by the TdT assay and flow cytometry 3.5 h after PDT. Data for untreated cells (**a**) and for doses killing 20% (**b**) and 85% (**c**) of the cells are presented here. Histograms (**i**) show the DNA content in the cells represented by the fluorescence from the DNA-specific dye Hoechst 33258. Contour plots (**ii** and **iii**) show the green fluoresence from fluorescein as a measure of free DNA ends *vs* the DNA content in cells stained in the presence (**ii**) and absence (**iii**) of TdT. Apoptotic cells were counted from regions R2, while regions R1 represent non-apoptotic cells. Signals from cells with less than half of the DNA content of G1 cells were not considered to avoid counting more than one cell fragment derived from a single cell. Without TdT being present, free DNA ends were not stained, as seen from regions R2 in (**iii**). The cell numbers represented by the contour lines increase by a factor of 2.5 between the lines.

by necrosis. To detect internucleosomal DNA cleavage, free DNA ends were stained with fluorescein by the TdT assay and total DNA was stained with the DNA-specific dye Hoechst 33258 before analysis by flow cytometry. Figure 1 shows flow cytometric data for V79 cells 3.5 h after ALA-PDT treatment with doses killing 20% and 85% of the cells, as well as for untreated control cells. On the contour plots (Figure 1ii), the intensity of green fluorescence from fluorescein representing free DNA ends is correlated to the DNA content in each single cell. Green background fluorescence was visible on control contour plots showing signals from cells stained unspecifically in the absence of TdT (Figure 1iii). Gates were placed such that signals from cells with only background staining, which were defined as non-apoptotic cells (regions R1), were separated from signals derived from cells with an increased intensity of green fluorescence owing to specific staining of free DNA ends (regions R2). The borders between regions R1 and R2 were positioned on the control contour plots (Figure 1iii) to the right of the background fluorescence such that regions R2 contained less than 1% of the signals on the control plots. The contour plots in Figure 1ii show that the number of free DNA ends was substantially increased by the treatment (regions R2 in Figure 1) in a large fraction of the cells. In Figure 1cii, 75% of the V79 cells treated with the highest ALA-PDT dose inducing 85% of cell death were counted from region R2. Regions R1 and R2 do not include signals with less than half of the DNA content of G1 cells. This is done to avoid counting more than one cell fragment derived from a single cell in our calculations. Nevertheless, the contour plots clearly show that the number of signals from cells with reduced DNA content was increased significantly (Figure 1cii, signals below regions R1+R2). Treatment of V79 cells with 5-ALA in the dark or with light alone did not have any cytotoxic effect nor induce an increased number of free DNA ends (data not shown). In the present study, the cells were incubated with 5-ALA in serum-free medium for 4 h before irradiation. This treatment in itself induced an increase in the number of free DNA ends in less than 5% of the cells. Furthermore, the lack of serum was not required for ALA-PDT to induce DNA cleavage. V79 cells incubated with 5-ALA in the presence of serum did undergo internucleosomal cleavage, although the light dose had to be increased by a factor of 10 to induce the same cytotoxic effect (data not shown).

Figure 2 shows flow cytometric data for WiDr cells treated with ALA-PDT doses killing 30% and 85% of the cells as well as for untreated cells. With the lowest dose and up to 27 h after ALA-PDT, no increase in the number of free DNA ends was induced (Figure 2ii, regions R2). One hour after ALA-PDT with the highest light dose, signals representing all levels of DNA content from small fragments

**Figure 2** ALA–PDT-induced necrosis in WiDr cells, as measured by the TdT assay and flow cytometry. The data for untreated cells (**a**) and doses killing 30% (**b** and **c**) and 85% (**d** and **e**) of the cells are shown here. As described in Figure 1, histograms (**i**) show the DNA content of the cells, while the contour plots show the DNA content *vs* the number of free DNA ends stained in the presence (**ii**) and absence (**iii**) of TdT. The cells were fixed 0.5 h (**b** and **d**), 1 h (**c**) and 27 h (**e**) after PDT. No significant numbers of cells with an increased number of free DNA ends were counted from regions R2.

**Figure 3** Internucleosomal DNA fragmentation induced by ALA-PDT in V79 cells but not in WiDr cells. DNA fragments extracted from untreated cells (−) and from cells being treated (+) with doses killing 85% of the cells (3.5 h after PDT), were separated by agarose gel electrophoresis and visualised with SYBR green under UV light. A DNA ladder of 185 bp fragments and its multiples appeared only for ALA-PDT-treated V79 cells and not in treated WiDr cells or in untreated control cells from both cell lines. The DNA ladder was shown in three separate experiments.

up to more than twice the diploid DNA content were obtained, as seen from the DNA histogram (Figure 2ei). The cells had developed sensitivity against trypsin and had to be scraped off for preparation of the samples.

To collect further support for the finding that ALA-PDT kills V79 and WiDr cells by different mechanisms, several other methods were applied. Figure 3 shows agarose gel electrophoresis of DNA fragments isolated from treated and untreated cells from both cell lines. Results are represented by samples prepared 3.5 h after treatment with ALA–PDT doses killing 85% of the cells. From ALA-PDT-treated V79 cells DNA fragments of approximately 185 bp and its multiples were isolated, forming the characteristic DNA ladder on the gel. On the contrary, for ALA-PDT-treated WiDr cells no DNA ladder was detectable. No DNA fragmentation was detectable in untreated control cells of either cell line.

In Figure 4, electron micrographs of ALA-PDT-treated and scraped off cells from both cell lines as well as of untreated control cells are shown. V79 cells fixed 2 h after ALA-PDT (Figure 4b) had the characteristic morphology of apoptotic cells. Membrane and organelle integrity was retained and membrane blebbing had occurred. The chromatin was condensed and formed a cap in the nuclear periphery, nucleoli were disintegrated and both cellular and nuclear fragmentation had occurred. Furthermore, in the light microscope a rounding of the V79 fibroblasts was observed within 0.5 h and, later on, the formation of membrane-bound cellular fragments (insert in Figure 4b). On the other hand, WiDr cells treated with different ALA-PDT doses did not show such morphological alterations. The electron micrograph of a WiDr cell 2 h after ALA-PDT (Figure 4d) is representative for these cells after treatment. The cell and its organelles appeared oedemic, the plasma membrane was partly destroyed and no chromatin condensation, nuclear fragmentation or formation of cell fragments was observed.

The fluorescence micrograph of ALA-PDT-treated V79 cells in Figure 5 visualises the localisation of free DNA ends stained with fluorescein by the TdT assay. On the inner side of the nuclear membrane, bright fluorescent spots were seen in two of the cells shown (Figure 5b, arrow). The rest of each single cell was stained weakly and homogeneously. ALA-PDT-treated WiDr cells stained similarly did not show bright fluorescent spots in their nuclei and only the weak, homogeneous fluorescence in the whole cells (data not shown).

**Figure 4** Electron micrographs of scraped off V79 (**a** and **b**) and WiDr cells (**c** and **d**). (**b**) and (**d**) show cells 2 h after ALA-PDT treatment with doses killing 85% of the cells, while those in (**a**) and (**c**) are untreated control cells. Chromatin condensation (cc), nuclear fragmentation (nf), membrane blebbing (bl) and apoptotic bodies (ab) are indicated by arrows. Scale bars = 1 $\mu$m. The insert in (**b**) gives an overview, showing a light micrograph of a semithin section of apoptotic and surviving cells, prepared as described in Materials and methods.

**Figure 5** Phase-contrast (**a**) and fluorescence micrographs (**b**) of V79 cells 3.5 h after ALA-PDT with a dose killing 85% of the cells. The fluorescence was derived from fluorescein which stains free DNA ends by the TdT assay (arrow). In addition, cells had a weak, homogeneous background staining with half of the intensity in the bright spots. The images were recorded by means of a CCD camera and the exposure time was 1 s.

*Quantification of the apoptotic fraction of ALA–PDT-treated V79 cells*

Apoptotic cells were defined as those cells having an increased number of free DNA ends, as measured by the TdT assay and flow cytometry. The apoptotic fraction was calculated as the ratio between signals counted from regions R2 on the contour plots of TdT-stained cells (see e.g. Figure 1cii) and signals in regions R1 + R2. The ratio of R2/R1 + R2 was corrected for the number of apoptotic cells in untreated control samples (0.3% for V79 cells and 0% for WiDr cells) and for cells with increased green fluorescence (regions R2) after staining in the absence of TdT (e.g. Figure 1iii).

The time dependence of the size of the fraction of apoptotic V79 cells was measured for several ALA–PDT doses. Figure 6 shows the apoptotic fractions of V79 cells treated with ALA–PDT doses killing 20%, 50%, 85% and 99% of the cells up to 5 h after treatment. All of the curves increased until a maximum was reached around 3–4 h after treatment after which the apoptotic fractions were reduced.

Figure 7 shows the apoptotic fractions measured 3.5 h after exposure to increasing ALA-PDT doses in comparison with the total cell death induced in two cell lines. The apoptotic fraction was largest for an ALA-PDT dose killing about 85% of the cells and was reduced when the dose killing 99% of the cells was used. The difference between total cell death and the apoptotic fraction was less than 20% after light doses inactivating less than 85% of the cells. For the highest light dose inactivating 99% of the cells, the difference between the two curves was 40%. For WiDr cells, no apoptotic cells were detected.

### Discussion

In the new modality of PDT reported by Malik and Lugaci (1987) and applied clinically by Kennedy *et al.* (1990), cells are killed by photoactivation of endogenous protoporphyrin IX which is accumulated when cells are exposed to the precursor 5-ALA. The mode of cell death induced by such a

**Figure 6** The time dependence of the apoptotic fraction measured by flow cytometry among ALA-PDT-treated V79 cells. The cells were treated with doses killing 20% (○), 50% (□), 85% (△) and 99% (▽) of the cells and fixed at different time intervals after PDT. The apoptotic fractions were calculated as the ratio between the number of apoptotic cells counted from regions R2 and all cells in regions R1 + R2, as described in Figure 1 and Results. The figure shows the results from representative experiments for 2–3 repeats where the maximal apoptotic fraction was reached between 3 and 4 h after PDT.

**Figure 7** Correlation between the apoptotic fraction (open symbols) and total cell death (filled symbols) of ALA-PDT-treated V79 cells (○ and ●; 0.1 mM 5-ALA) and WiDr cells (△ and ▲; 1 mM 5-ALA). Cell death was measured 24 h after ALA–PDT by the cell survival assay described in Materials and methods, while the apoptotic fractions were determined from flow cytometric measurements 3.5 h after ALA–PDT as described in Figures 1 and 6. The bars represent the range for 2–3 experiments.

treatment was studied in two cell lines, V79 and WiDr cells. ALA-PDT-treated V79 cells developed characteristic features of cells undergoing apoptosis. Both the typical morphology (Kerr *et al.*, 1972) and DNA fragmentation at internucleosomal sites were shown (Figures 3 and 4). Another evidence of apoptosis is the reduction of the DNA content owing to leakage of low molecular weight DNA from the cells during the staining procedure, shown by a decrease in the fluoresence from the DNA-staining dye Hoechst 33258 (Darzynkiewicz *et al.*, 1994; Figure 1cii, signals below regions R1 + R2). This reduction of the DNA content could also be caused by fragmentation of nuclei and cells. Therefore we did not include signals outside regions R1 and R2 in our calculations to avoid counting several cell fragments derived from the same cell. Furthermore, a highly specific assay of apoptosis by flow cytometry is based on fluorescent staining of free DNA ends with fluorescein after incorporation of biotinylated dUTP in the presence of TdT (Gorczyca *et al.*, 1993). A substantial incorporation of biotin-dUTP was measured in the prefixed ALA-PDT-treated V79 cells (Figure 1). PDT is known to induce primary DNA strand breaks (Moan *et al.*, 1980; Noodt *et al.*, 1993). An important question is, therefore, whether the labelled free DNA ends were produced by direct DNA damage. Meanwhile, PDT-induced primary DNA strand breaks are repaired within 0.5 h (Kvam and Moan, 1990) while the maximal incorporation of biotin–dUTP was measured 3–4 h after PDT (Figure 6). Measurements after 0.5 h gave an absolute apoptotic fraction of only less than 10% of the cells, as distinguished by the TdT assay, even after treatment with doses killing about 85% of the cells (Figure 6). Furthermore, the number of directly formed, primary DNA strand breaks in PDT is too low to give a strong enough fluorescent signal to be distinguished by the flow cytometric assay. PDT induces one primary DNA strand break per 155 kb at most (Kvam *et al.*, 1990), while spacing of internucleosomal cleavage sites is 180 bp, making a difference of about a factor of $10^3$. Dividing the highest intensity of green fluorescence obtained by the flow cytometric assay of apoptosis (Figure 1) by a factor of $10^3$ would result in a signal with an intensity much lower than the background. Consequently, signals from cells with maximal primary DNA strand breakage induced by PDT would be localised in region R1 and not in region R2 from where the apoptotic cells were counted. The ladder of DNA fragments of oligonucleosomal sizes (Figure 3) provides further evidence that primary DNA damage is of no importance in this context of apoptotic assays.

The WiDr cells did not show the typical morphology of apoptotic cells and did not retain their plasma membrane integrity (Figures 4 and 2ei). Digestion of the plasma membrane and disappearance of the cells from the samples during trypsinisation are considered as a probe for necrotic cells (Darzynkiewicz *et al.*, 1994). Our results indicate that necrosis can be studied on cells that are scraped off from the culture flasks, while apoptosis can be assayed on attached cells either trypsinated or scaped off. Necrosis in WiDr cells was further evident from flow cytometric measurements of biotin-dUTP incorporation. No increase was measured either after ALA–PDT with a variety of doses (Figures 2 and 7) or at different time intervals after the treatment (Figures 2 and 6).

Cell-specific induction of apoptosis is normally explained by differences between cell types in their ability to receive or to respond to cell death signals. A cell with lack or gain of a functional gene product required for apoptosis is assumed to be killed by necrosis in a treatment-dependent manner. To initiate apoptosis, one or more of several parallel pathways have to be triggered by a stimulus leading to the activation of endonucleases, which is viewed as a typical end point in apoptosis (reviewed by Martin *et al.*, 1994). The pathway transmitting the apoptotic signals in ALA–PDT does not seems to involve protein synthesis since apoptosis was observed within less than an hour and down to minutes, as shown earlier (Agarwal *et al.*, 1991; Oleinick *et al.*, 1993) and in our experiments (Figure 6). In PDT, activation of endonucleases has been stimulated directly by the influx of free $Ca^{2+}$, a known trigger of apoptosis, and by inhibition of the poly(ADP-ribosyl)polymerase (Ben-Hur *et al.*, 1991; Penning *et al.*, 1994). However, DNA fragmentation elicited in this way did not seem to result in the characteristic DNA

ladder obtained by gel electrophoresis (Penning et al., 1994). Although PDT in general seems to induce an increase in intracellular free $Ca^{2+}$, activation of internucleosomal DNA fragmentation is clearly cell line dependent. Cell-specific induction of apoptosis has also been shown earlier since human non-small-lung carcinoma cells did not undergo apoptosis in response to PDT with Photofrin II, whereas human prostate carcinoma cells and rat mammary carcinoma cells did (He et al., 1994). Thus, no direct activation of endonucleases seems to be responsible for the apoptotic process. In WiDr cells, apoptosis could not be induced by either ALA-PDT or by X-irradiation, in contrast to V79 cells which responded within 48 h (data not shown). Therefore, the pathway leading to apoptosis in WiDr cells seems to be inhibited at a late common step.

An interesting consideration in PDT is whether photosensitisers inducing apoptosis do so by different pathways although they are assumed to act mainly via singlet oxygen (Moan and Berg, 1991). In contrast to 5-ALA in our experiments, AlPc has been reported not to induce apoptosis in V79 cells (Oleinick et al., 1993). An explanation for this contradiction may be found in the localisation of the photosensitisers, since singlet oxygen acts only in its immediate proximity (Weishaupt et al., 1976; Moan et al., 1979; Moan and Berg, 1991). Protoporphyrin IX, the endogenous product synthesised in the presence of 5-ALA, is localised in mitochondrial membranes and in the plasma membrane in V79 cells. AlPc may be localised differently and thereby be too distant from sensors activating the apoptotic pathway. Another explanation may be that AlPc, as well as other phthalocyanine derivatives, also acts through other relative oxygen species (Ben-Hur and Rosenthal, 1985; Gantchev, 1992; Zaidi et al., 1993). Thus, in PDT there may be an additional requirement for apoptosis to be induced in a certain cell type. Not only do the responding pathways have to be functional, but their sensors also have to be within a short distance of the photosensitiser within the cells.

Cells undergo apoptosis in response to relatively low doses of various treatments (Lennon et al., 1991) whereas larger doses do induce necrosis. For V79 cells, the response curve for induction of apoptosis after ALA-PDT has an optimum for light doses inducing about 80–90% of total cell death (Figure 7). For higher light doses, the fraction of apoptotic cells is reduced and the necrotic fraction increased. Furthermore, the time interval between induction and quantification of apoptosis has to be considered here. The maximal apoptotic fraction was measured during a narrow time span between the expression of apoptotic features and the onset of cell fragmentation (Figure 6). This effect seemed to be more pronounced for higher light doses (Figure 6, see curve for 99% of cell death), probably owing to a more extended fragmentation and formation of small apoptotic bodies. If the DNA content was less than half of the DNA content of G1 cells, signals were localised outside regions R1 and R2 and are not included in our calculations. They may also have been lost during the staining procedure to a greater extent than larger apoptotic bodies. Therefore, for quantification of the apoptotic fraction, both the time interval after treatment and the dose applied are important parameters. Quantification is of importance in studies on the mode of cell death induced by a treatment and when aiming at the induction apoptosis for specific and complete eradication of cancer cells.

### Abbreviations

PDT, photodynamic treatment; 5-ALA, 5-aminolaevulinic acid; TdT, terminal deoxynucleotide transferase.

### Acknowledgements

We are grateful for expert technical assistance from Torstein Schjerven on data technology handling, from Inga Finseth on electron microscopy and from Torild Aasen on flow cytometry. This work was supported by the Research Council of Norway and the Norwegian Cancer Society.

### References

AGARWAL ML, CLAY ME, HARVEY EJ, EVANS HH, ANTUNEZ AR AND OLEINICK NL. (1991). Photodynamic therapy induces rapid cell death by apoptosis in L5178Y mouse lymphoma cells. *Cancer Res.*, **51**, 5993–5996.

ARENDS MJ, MORRIS RG AND WYLLIE AH. (1990). Apoptosis–The role of the endonuclease. *Am. J. Pathol.*, **136**, 593–608.

BEN-HUR E AND ROSENTHAL I. (1985). The phthalocyanines: a new class of mammalian cell photosensitizers with a potential for cancer phototherapy. *Int. J. Radiat. Biol.*, **47**, 145–147.

BEN-HUR E, DUBBELMAN TMAR AND VAN STEVENINCK J. (1991). Phthalocyanine-induced photodynamic changes of cytoplasmic free calcium in Chinese hamster cells. *Photochem. Photobiol.*, **54**, 163–166.

BERG K, HOVIG E AND MOAN J. (1988). Sister chromatid exchanges induced by photodynamic treatment of cells in the presence of Photofrin II, aluminum phthalocyanine tetrasulfonate and tetra(3-hydroxy-phenyl)porphyrin. In *Light in Biology and Medicine*. Vol.1, Douglas J, Dall'Aqua F and Moan J, (Eds), pp. 95–103. Plenum Press: New York.

BERG K, MADSLIEN K, BOMMER JC, OFTEBRO R, WINKLEMAN JW AND MOAN J. (1991). Light induced relocalization of sulfonated meso-tetraphenylporphines in NHIK 3025 cells and effects of dose fractionation. *Photochem. Photobiol.*, **53**, 203–210.

BERG K, LUKSIENE Z, MOAN J AND MA L. (1995). Combined treatment of ionizing radiation and photosensitization by 5-aminolevulinic acid-induced protoporphyrin IX. *Radiation Res.*, **142**, 340–346.

BERG K, ANHOLT H, BECH Ø AND MOAN J. (1996). Inhibition of ferrochelatase activity in 5-aminolaevulinic acid-treated cells by iron chelators. (submitted).

CARSON DA AND RIBERIO JM. (1993). Apoptosis and disease. *Lancet*, **341**, 1251–1254.

DAILEY HA AND SMITH A. (1984). Differential interaction of porphyrins used in photoradiation therapy with ferrochelatase. *Biochem. J.*, **223**, 441–445.

DARZYNKIEWICZ Z, LI X AND GONG J. (1994). Assays of cell viability: discrimination of cells dying by apoptosis. *Methods Cell Biol.*, **41**, 15–38.

DOUGHERTY TJ. (1993). Photodynamic therapy. *Photochem. Photobiol.*, **58**, 895–900.

DOUGHERTY TJ, KAUFMAN JE, GOLDFARB A, WEISHAUPT KR, BOYLE DG AND MITTLEMAN A. (1978). Photoradiation therapy for the treatment of malignant tumors. *Cancer Res.*, **38**, 2638–2635.

FISHER DE. (1994). Apoptosis in cancer therapy: crossing the threshold. *Cell*, **78**, 539–542.

GANTCHEV TG. (1992). Spin-trapping of free radicals during phthalocyanine photosensitization of lymphoma cells *in vitro*. *Cancer Biochem. Biophys.*, **13**, 103–111.

GAULLIER J-M, GÈZE M, SANTUS R, SA E MELO T, MAZIÈRE J-C, BAZIN M, MORLIÈRE P AND DUBERTRET L. (1995). Subcellular localisation of and photosensitization by protoporphyrin IX in human keratinocytes and fibroblasts cultivated with 5-aminolevulinic acid. *Photochem. Photobiol.*, **62**, 114–122.

GORCZYCA W, GONG H AND DARZYNKIEWICZ Z. (1993). Detection of DNA strand breaks in individual apoptotic cells by the *in situ* terminal deoxynucleotidyl transferase and nick translation assays. *Cancer Res.*, **53**, 1945–1951.

HE X-Y, SIKES RA, THOMSEN S, CHUNG LWK AND JACQUES SL. (1994). Photodynamic therapy with Photofrin II induces programmed cell death in carcinoma cell lines. *Photochem. Photobiol.*, **59**, 468–473.

HERRMANN M, LORENZ H-M, VOLL R, GRUNKE M, WOITH W AND KALDEN JR. (1994). A rapid and simple method for the isolation of apoptotic DNA fragments. *Nucleic Acids Res.*, **24**, 5506–5507.

KENNEDY JC, POTTIER RH AND PROSS DC. (1990). Photodynamic therapy with endogenous protoporphyrin IX. Basic principles and present clinical experience. *J. Photochem. Photobiol. B:Biol.*, **6**, 143–148.

KERR JFR, WYLLIE AH AND CURRIE AH. (1972). Apoptosis: a basic biological phenomenon with wide-ranging implications in tissue kinetics. *Br. J. Cancer*, **26,** 239–257.

KVAM E AND MOAN J. (1990). A comparison of three photosensitizers with respect to efficiency of cell inactivation, fluorescence quantum yield and DNA strand breaks. *Photochem. Photobiol.*, **52,** 769–773.

KVAM E, STOKKE T AND MOAN J. (1990). The lengths of DNA fragments light-induced in the presence of a photosensitizer localized at the nuclear membrane of human cells. *Biochim. Biophys. Acta*, **1049,** 33–37.

LENNON SV, MARTIN SJ AND COTTER TG. (1991). Dose-dependent induction of apoptosis in human tumour cell lines by widely diverging stimuli. *Cell Prolif.*, **24,** 203–214.

MCDOWELL EM AND TRUMP BF. (1976). Histologic fixatives suitable for diagnostic light and electron microscopy. *Arch. Pathol. Lab. Med.*, **100,** 405–414.

MALIK Z AND LUGACI H. (1987). Destruction of erythroleukaemic cells by photoactivation of endogenous porphyrins. *Br. J. Cancer*, **56,** 589–595.

MARTIN SJ, GREEN DR AND COTTER TG. (1994). Dicing with death: dissecting the components of the apoptosis machinery. *Trends Biol. Sci.*, **19,** 26–30.

MOAN J AND BERG K. (1991). The photodegradation of porphyrins in cells can be used to estimate the lifetime of singlet oxygen. *Photochem. Photobiol.*, **53,** 549–553.

MOAN J, PETTERSEN EO AND CHRISTENSEN T. (1979). The mechanisms of photodynamic inactivation of human cells *in vitro* in the presence of heamatoporphyrin. *Br. J. Cancer*, **39,** 398–407.

MOAN J, WAKSVIK H AND CHRISTENSEN T. (1980). DNA single-strand breaks and sister chromatid exchanges induced by treatment with hematoporphyrin and light or by X-rays in human NHIK 3025 cells. *Cancer Res.*, **40,** 2915–2918.

MOAN J, HØVIK B AND SOMMER S. (1984). A device to determine fluence-response curves for photoinactivation of cells *in vitro*. *Photobiochem. Photobiophys.*, **8,** 11–17.

MOLLENHAUER HH. (1964). Plastic embedding mixtures for use in electron microscopy. *Stain. Technol.*, **39,** 111–114.

NOGUCHI P, WALLACE R, JOHNSON J, EARLEY EM, O'BRIEN S, FERRONE S, PELLEGRINO MA, MILSTEIN J, NEEDY C, BROWNE W AND PETRICIANI J. (1979). Characterization of WiDr: a human colon carcinoma cell line. *In Vitro*, **15,** 401–408.

NOODT BB, KVAM E, STEEN HB AND MOAN J. (1993). Primary DNA damage, HPRT mutation and cell inactivation photoinduced with various sensitizers in V79 cells. *Photochem. Photobiol.*, **58,** 541–547.

OLEINICK NL, AGARWAL ML, BERGER NA, BERGER S, CHENG M-F, CHATTERJEE S, HE J, KENNEY ME, LARKIN HE, MUKHTAR H, RIHTER BD AND ZAIDI SIA. (1993). Signal transduction and metabolic changes during tumor cell apoptosis following phthalocyanine-sensitized photodynamic therapy. *Society of Photo-Optical Instrumentation Engineers*, **1881,** 252–261.

PENNING LC, LAGERBERG JWM, VAN DIERENDONCK, CORNELISSE CJ, DUBBELMAN TMAR AND VAN STEVENINCK J. (1994). The role of DNA damage and inhibition of poly(ADP-ribosyl)ation in loss of clonogenicity of murine L929 fibroblasts, caused by photodynamically induced oxidative stress. *Cancer Res.*, **54,** 5561–5567.

WEISHAUPT KR, GOMER CJ AND DOUGHERTY TJ. (1976). Identification of singlet oxygen as the cytotoxic agent in photoinactivation of a murine tumor. *Cancer Res.*, **36,** 2326–2329.

WYLLIE AH. (1993). Apoptosis (The 1992 Frank Rose memorial lecture). *Br. J. Cancer*, **67,** 205–208.

ZAIDI SIA, AGARWAL R, EICHLER G, RIHTER BD, KENNEY ME AND MUKHTAR H. (1993). Photodynamic effects of new silicon phthalocyanines: *In vitro* studies utilizing rat hepatic microsomes and human erythrocyte ghosts as model membrane sources. *Photochem. Photobiol.*, **58,** 204–210.